# Robot-Assisted Epiretinal Membrane Peeling: A Prospective Assessment of Pre- and Intra-Operative Times and of Surgeons’ Subjective Perceptions

**DOI:** 10.3390/jcm12082768

**Published:** 2023-04-07

**Authors:** Ferhat Turgut, Gábor Márk Somfai, Florian M. Heussen, Alexander Eberle, Marc D. de Smet, Matthias D. Becker

**Affiliations:** 1 Department of Ophthalmology, Stadtspital Zurich, 8063 Zurich, Switzerland; ferhat.turgut@stadtspital.ch (F.T.); gabor.somfai@stadtspital.ch (G.M.S.); florian.heussen@insel.ch (F.M.H.); alexander.eberle@stadtspital.ch (A.E.); 2 Werner H. Spross Foundation for the Advancement of Research and Teaching in Ophthalmology, 8063 Zurich, Switzerland; 3 Department of Ophthalmology, Semmelweis University, 1085 Budapest, Hungary; 4 Department of Ophthalmology, Inselspital, University Hospital Bern, 3010 Bern, Switzerland; 5 Helvetia Retina Associates, 1005 Lausanne, Switzerland; marcdesmet@preceyes.nl; 6 New York Eye and Ear Infirmary of Mt Sinai, Icahn School of Medicine, New York, NY 10029, USA; 7 Department of Ophthalmology, University of Heidelberg, 69117 Heidelberg, Germany

**Keywords:** epiretinal membrane, membrane peeling, vitreoretinal surgery, robot-assisted membrane peeling, robotic surgery, operative times, surgeon satisfaction, surgical precision

## Abstract

Purpose: The Preceyes Surgical System (PSS) is a robotic assistive device that may enhance surgical precision. This study assessed pre- and intra-operative times and surgeons’ perceptions of robot-assisted epiretinal membrane peeling (RA-MP). Methods: We analyzed the time requirement of three main tasks: the preparation of the PSS (I), patient preparation (II), and surgery (III). Following surgery, the surgeons were asked questions about their experience. Results: RA-MP was performed in nine eyes of nine patients. Task I required an average time of 12.3 min, initially taking 15 min but decreasing to 6 min in the last surgery. Task II showed a mean time of 47.2 (range of 36–65) min. Task III had a mean time of 72.4 (range of 57–100) min. A mean time of 27.9 (range of 9–46) min was necessary for RA-MP. The responses to the questionnaire revealed a trend towards increasing ease and reduced stress as familiarity with the PSS increased. Conclusions: A substantial reduction in pre- and intra-operative times, decreasing to a total of 115 min, was demonstrated. RA-MP was positively anticipated by the surgeons and led to no hand or arm strain while being more complex than manual MP.

## 1. Introduction

Robotic surgery is a field of medicine that has gained significant attention and traction in recent years. It involves the use of surgical robots to perform various surgical procedures with increased control, precision, and flexibility compared to traditional surgical methods. The history of robotic surgery dates to the late 1980s and early 1990s, when the first robotic surgical systems were developed and introduced [1]. Since then, the field has undergone tremendous growth and evolution, leading to a wide range of advancements and benefits for both patients and surgeons.

One of the key advantages of robotic surgery is its ability to provide enhanced dexterity and precision in delicate surgical procedures, especially in minimally invasive surgery involving sensitive tissue. These advantages of robotic surgery, especially its high precision, offer a great opportunity to use it in ophthalmic surgery, e.g., in the delicate process of epiretinal membrane peeling during retinal surgery, among others. An epiretinal membrane (ERM) is a thin layer of about 30–90 micrometers of tissue that forms on the inner surface of the retina [2]. The layer can be caused by a variety of factors, including aging, injuries, previous intraocular surgery, and retinal vascular diseases, and it may lead to a range of visual symptoms, including blurred or distorted vision [3]. A vitrectomy with the peeling of the ERM and/or of the ILM is performed as a treatment.

The Preceyes Surgical System (PSS; Carl Zeiss Meditec AG, Jena, Germany) is a robotic assistive device for vitreoretinal surgery [4]. In 2019, the device obtained CE certification, which allows its use for vitreoretinal surgery in the European Economic Area (EEA). One of the key advantages of the PSS is its ability to provide a high level of precision and accuracy during challenging surgical maneuvers. It was developed to overcome the shortcomings of manual surgery in limited confined spaces [5]. Even though the duration of surgery with the PSS can be longer at the beginning than manual surgery, its ability to accurately replicate the movements of the surgeon’s hands offers the potential to minimize retinal trauma and improve outcomes [6].

The efficient integration of surgical robots into surgical procedures can enhance surgical precision and accuracy; however, it requires careful planning, training, and preparation. In order to evaluate the usability of the PSS in the operating room (OR) and its interference with the OR staff and OR routine, we assessed the times necessary for the preoperative preparation and for the surgical intervention itself for a comparison to manually performed surgery. Furthermore, a questionnaire was filled out by the surgeons after each surgery to evaluate the subjective perception of robot-assisted epiretinal membrane peeling (RA-MP).

## 2. Patients and Methods

A total of nine eyes out of nine patients received RA-MP with the PSS in November 2022 at the Department of Ophthalmology, Stadtspital Zurich, Zurich, Switzerland. All patients were extensively informed about the procedure, possible complications, and the functionality of the PSS. The patients signed an informed consent for the surgery and agreed to the use of their data for research purposes. The local ethics committee confirmed in a declaration of responsibility that the publication of data concerning the pre- and peri-operative times, as well as the surgeon’s questionnaire, does not fall within the scope of the Swiss Human Research Act and, therefore, does not require the approval of the Cantonal Ethics Committee for its implementation.

**Figure 1:**
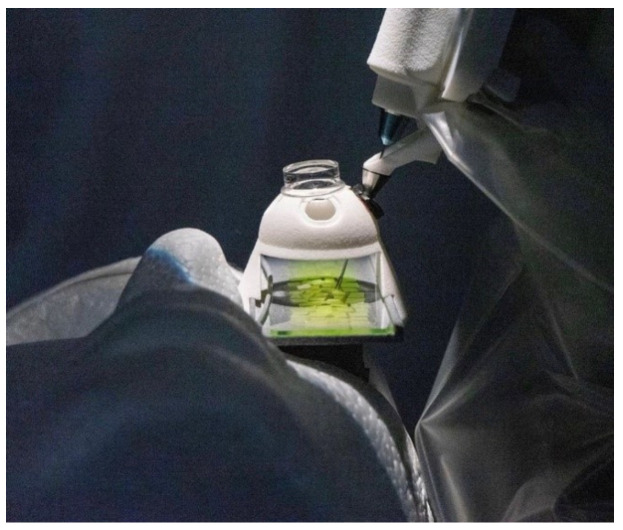
Practical training simulating epiretinal membrane peeling in order to familiarize the surgeon with the functionality of the Preceyes Surgical System (PSS). The phantom eye contained a gelatin layer with superficial embedded thin paper to be peeled during the training.

Two experienced vitreoretinal surgeons (M.D.B. and F.M.H.) received intensive training for the operation of the PSS. In the theoretical part, all functions and safety measures of the PSS were explained and demonstrated by the PSS team. For the practical part, a phantom eye containing a gelatin layer with a superficial embedded thin paper was provided to exercise epiretinal membrane peeling (Figure 1). The phantom eye was used bilaterally to familiarize the surgeon with using the motion controller in both hands.

**Figure 2:**
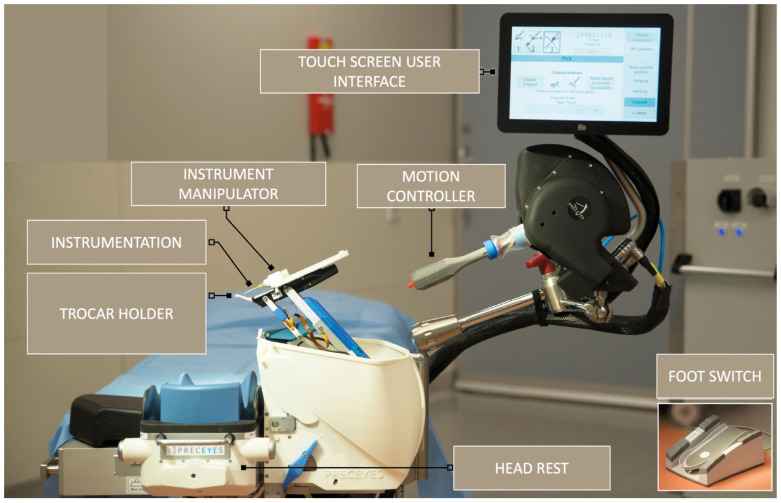
An overview of the Preceyes Surgical System (PSS). The system is based on a telemanipulation system: the motion controller is used as a joystick and translates rough movements into fine movements of the intraocular instrument. The instrument is attached to the instrument manipulator and enters the globe via a trocar. A touch screen is used to switch between different modules. The foot switch can be used to open and close the forceps pneumatically (image courtesy of Carl Zeiss Meditec AG, Jena, Germany).

The motion controller is handled as a joystick to telemanipulate the intraocular instrument held by the instrument manipulator (Figure 2). Furthermore, two surgical nurses attended special training that addressed the operating principles and the draping of the PSS.

A comprehensive clinical examination of each patient was performed before the surgery, including a best-corrected visual acuity examination, an intraocular pressure examination, a slit-lamp examination, fundus examination, and spectral-domain optical coherence tomography (Heidelberg Engineering, Inc., Heidelberg, Germany) of the macula.

The indications for the surgeries were visual impairment due to the epiretinal membrane (n = 7), a full-thickness macular hole (n = 1), and myopic macular schisis (n = 1). The surgeries were performed under general anesthesia. In four cases, the intervention was combined with phacoemulsification and the implantation of an intraocular lens.

The first step of surgery was phacoemulsification and intraocular lens implantation in four phakic eyes, while the surgery in five pseudophakic eyes started with a conventional 23-gauge vitrectomy with the vitrectomy system EVA (DORC, Zuidland, the Netherlands): A special cone-shaped trocar (Alcon Laboratories, Inc., Fort Worth, TX, USA), which provided the connection point to the trocar holder of the instrument manipulator, was inserted temporally. Furthermore, chandelier twin lights (DORC, Zuidland, The Netherlands) were inserted superiorly. A core and peripheral vitrectomy were performed with the induction of a posterior vitreous detachment as necessary. After vitrectomy, staining with MembraneBlue-Dual (DORC, Zuidland, the Netherlands) was applied and removed. The instrument manipulator was connected to the trocar by a special movement sequence of the motion controller. After successfully connecting the PSS to the patient’s eye, the surgeon robotically inserted the 23-gauge end-gripping forceps (Optico Ltd., Letchworth Garden City, Great Britain) through the trocar into the globe. A flap of the membrane was initiated, and RA-MP was performed. Finally, a fluid–air exchange was performed in seven eyes, while in two eyes, a silicon oil tamponade was applied.

On the day of surgery, the times for the preoperative preparation and for all the surgical steps were assessed in minutes (Table 1). Three main tasks were defined: the preparation of the PSS (task I), patient preparation (task II), and surgical intervention (task III). After the installation of the PSS at the headrest, the timing of the preparation (task I) started with the draping of certain parts of the PSS (instrument manipulator, trocar holder, motion controller, and cables). An extra draping was provided on the instrument manipulator to guarantee sterility during the next task.

**Figure 3:**
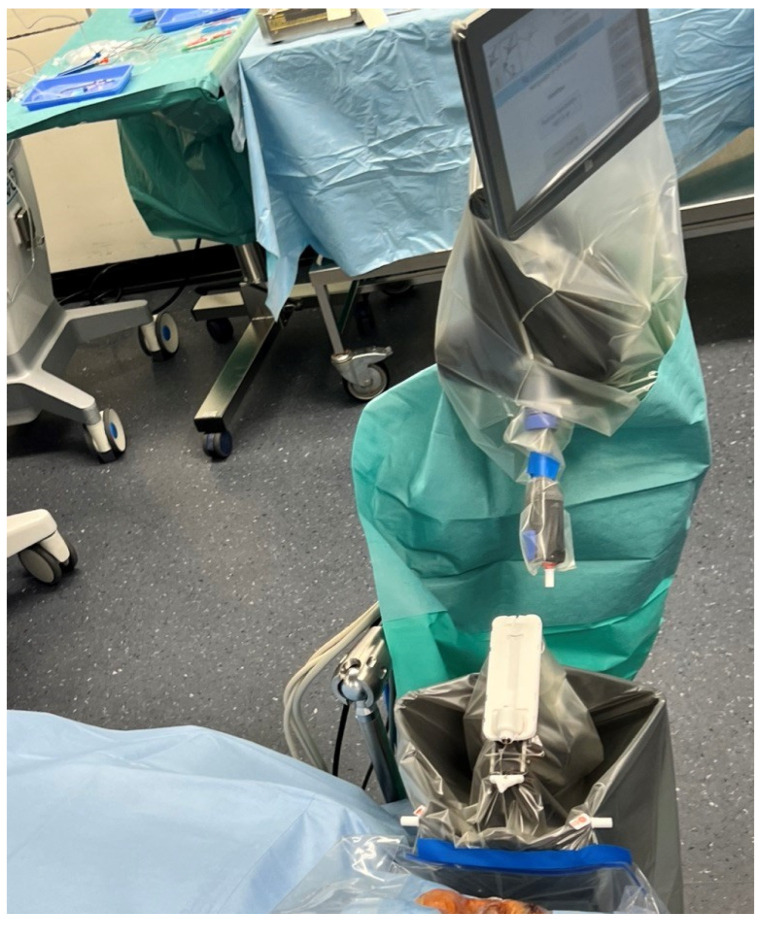
The draping of the PSS contains the instrument manipulator, trocar holder, motion controller, and cables. The extra draping, which maintains the sterility of the instrument manipulator during the introduction of anesthesia, has already been removed.

The next stage of patient preparation (task II) started following the patient’s entrance into the OR. After the induction of general anesthesia, a reachability check of the trocar holder and the patient was performed. When necessary, the patient’s head was repositioned in case the reach was not appropriate. The final preparatory steps for standard eye surgery were then carried out (Figure 3).

The surgical intervention (task III) started with the insertion of the first trocar and ended with the removal of the last trocar. Several subtasks were defined, such as the trocar placement, the vitrectomy, the placement of the chandelier twin light, and the membrane peeling. The peeling procedure was the most critical subtask of the surgical intervention. As soon as the instrument manipulator was moved to the trocar (connection robot and trocar), the subtask started. After the successful connection of the robot and the trocar, the forceps was inserted into the eye and moved toward the retina. The subtask of the membrane peeling started with the first grasp and ended with the removal of the forceps.

Following surgery, the surgeons filled out a questionnaire with the following questions:

- Use of the robot for peeling is easier compared to manual peeling.
- Executing peeling with the robot was less stressful compared to manual surgery.
- During the surgery, I experienced no hand or arm strain.
- Further use of the robot desired.

The answer options were the following: strongly disagree, disagree, neutral, agree, and strongly agree.

Due to the low number of cases, no statistical analysis was performed, and only descriptive data are reported.

## 3. Results

Surgeon F.M.H. performed four surgeries, while surgeon M.D.B. performed five surgeries (Table 2).

The preparation of the PSS (task I) required a mean time of 12.3 min and demonstrated a learning curve of a maximum of 15 min decreasing to 6 min at the end of the study period (Table 3). The reduction in draping time also contributed to the continuous improvement of the protocol: the draping of the touch screen and the motion controller was initially performed before the patient was in anesthesia and, therefore, required an extra cover of draping to maintain sterility during introduction of anesthesia; after the sixth case the motion controller and the touch screen were draped after anesthesia and therefore did not need an extra draping to protect sterility.

Patient preparation (task II) demonstrated a mean time of 47.2 min, with a range from 36 to 65 min. The introduction of general anesthesia had a mean time of 25.6 min, ranging from 9 to 55 min. Anesthesia is routinely introduced in the anesthetic room, which is located at the entrance of the OR. However, this routine was not possible for RA-MP due to the connection of the sterile draped PSS to the OR table. Before the introduction of general anesthesia, a good position of the patient’s head on the head cushion had to be confirmed. The patient’s final preparation, such as eye draping, took a mean time of 14.4 (range 2 to 21) min.

Surgical intervention (task III) had a mean time of 72.4 min, with a range from 57 to 100 min. Vitrectomy had a mean duration of 7.9 min, ranging from 4 to 13 min. The insertion of the chandelier twin light (mean 3.4 min, range of 3−5 min) intended to avoid movements of the globe during the peeling procedure; however, to facilitate retinal visualization, an intraocular light source was additionally used through one of the remaining trocars. RA-MP was performed in a mean time of 27.9 min, with a range of 9 to 46 min. When the forceps were removed before finalizing the membrane peeling (e.g., in cases of repeated staining), the time for a second connection of the robot and trocar was tracked and added to task III’s time. Several snapshots, as well as volume scans, of intraoperative optical coherence tomography (ARTEVO, Carl Zeiss Carl Zeiss Meditec AG, Jena, Germany) were acquired in each surgery at the moment of membrane grasp or during the peeling procedure, the duration of which was also added to the subtask.

The time of the whole procedure (start point: draping; end point: removal of the last trocar) had a mean of 132 (range of 115 to 172) min. Since four phakic eyes received cataract surgery in the same procedure, the time required (performing paracentesis until implanting intraocular lens) was excluded.

**Figure 4:**
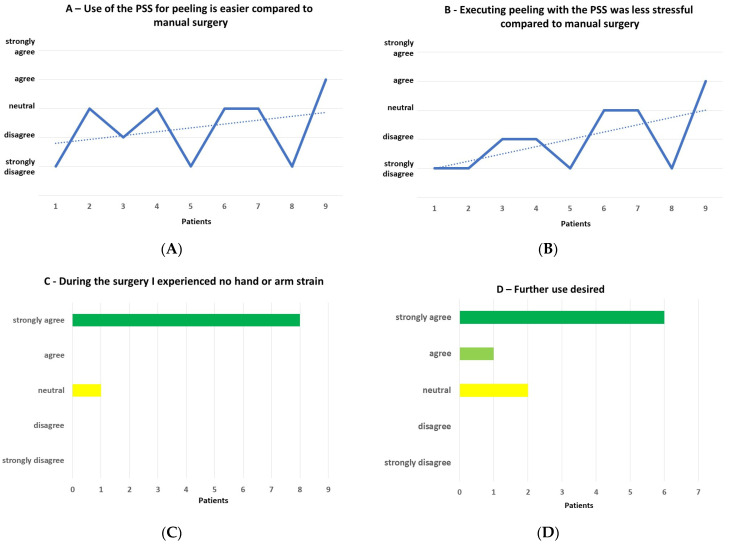
(**A**) Use of the PSS for peeling is easier compared to manual peeling. (**B**) Executing peeling with the PSS was less stressful compared to manual surgery. While the solid lines connect the responses after each case, the dashed lines present a trend line indicating easier handling and less stress over time. (**C**) During the surgery, I experienced no hand or arm strain. (**D**) Further use desired.

The responses to the questionnaire were as follows (Figure 4A–D):

“Use of the robot for peeling is easier compared to manual peeling.”

- Strongly disagree = 3, disagree = 1, neutral = 4, agree = 1, strongly agree = 0.

“Executing peeling with the robot was less stressful compared to manual surgery.”

- Strongly disagree = 4, disagree = 2, neutral = 2, agree = 1, strongly agree = 0.

“During the surgery, I experienced no hand or arm strain.”

- Strongly disagree = 0, disagree = 0, neutral = 1, agree = 0, strongly agree = 8.

“Further use of the robot desired.”

- Strongly disagree = 0, disagree = 0, neutral = 2, agree = 1, strongly agree = 6.

There was a trend towards increasing ease and less stress with time.

## 4. Discussion

Robotic surgery is a rapidly developing field that offers new opportunities for minimally invasive procedures and improved surgical outcomes. The use of robotic assistance in surgery dates to the late 1980s, with the da Vinci surgical system being FDA-approved in the year 2000 [1]. Since then, it has become one of the most popular robotic platforms in the world, paving the way for new robotic devices. However, before establishing a new potential robotic device in the OR routine, certain questions concerning safety, as well as efficiency, must be answered conscientiously.

Studies have demonstrated that the use of robot-assisted surgery often results in longer surgical times than traditional laparoscopic surgery, which may potentially lead to less surgeries per day [7,8]. A meta-analysis comparing the surgical times of robot-assisted gastrectomies to those performed manually found that robot-assisted procedures were 1.06–1.46 times longer than manual procedures [9].

The learning curve in surgical procedures is an ongoing process, and improvement in various tasks can significantly impact the efficiency of the surgical team. In this case of the PSS, the surgical nurses successfully reduced the draping time from 15 to 6 min through intensive training. After the first surgeries, they were able to perform the draping independently, further improving the efficiency of the procedure. To further increase workflow efficiency, it is recommended to introduce anesthesia in the anesthetic room. As general anesthesia is required for this surgical procedure, connecting the trocar and the robot in the anesthetic room could save time. The PSS installation could also be carried out after the anesthetic procedure, maybe even with a mobile version of the PSS. This way, different tasks can be performed simultaneously, and the surgical time needed can be significantly reduced.

Our study demonstrated a mean time of 27.9 (range of 9–46) min for RA-MP, while a manually performed MP takes a duration of 5−10 min. When adding the time needed for switching to RA-MP, the duration of the procedure may be even longer (in total, a mean time of 41.8 min, with a range of 22–66 min). An intensive training phase prepared the surgeons for the connection of IM to the trocar and RA-MP, using an artificial eye model; however, each surgeon had only four to five eyes to perform surgery on. Consequently, the potential learning curve could not be fully exhausted. To improve the learning curve, further surgeries are required. An exciting aspect is the surgery duration for RA-MP in the case of novice surgeons. According to Jacobsen et al., there was no significant difference between the lengths of the learning curves for RA-MP compared to those for manual surgery [10]. However, RA-MP was more precise and associated with less tissue damage.

An important issue in the above aspect is the cost of robots in surgery. The use of the DaVinci robot is often associated with surgical costs higher than those of traditional laparoscopic surgery. The costs of purchasing and maintaining the robotic system, as well as the additional training required for surgeons, contribute to the higher expenses [11]. Despite these higher costs, there is evidence to suggest that the long-term use of the DaVinci robot can result in a reduction in medical costs. This is due to the reduced risk of complications and improved patient outcomes, which can lead to shorter hospital stays and a lower need for follow-up medical interventions [7]. Another factor that could reduce the costs of robotic surgery in the future is competition. The costs of robotic surgical systems and their associated instrumentation and maintenance could begin to decline with greater competition and increased usage in the market [12].

A longer surgical duration will probably lead to less surgeries per day; still, one should consider multiple aspects here: even though the surgical times might be longer in the beginning, one of the most significant benefits of the DaVinci robot is improved patient-reported outcomes. Patients who undergo surgery using the DaVinci robot experience less pain and a faster recovery time than those who undergo traditional laparoscopic surgery [13]. The robot’s precise movements can lead to reduced tissue trauma, which can result in less postoperative pain and a quicker return to normal activities [6,14]. Moreover, in the case of the PSS, the most valuable advantage is the precision of the instrument movement, which has the potential to create less (micro-) trauma to the retina when grasping the membrane [15,16]. Whether RA-MP induces less retinal trauma than manually performed peeling must be further investigated. In the case of a significant difference between these techniques, a longer duration for surgery should be accepted in favor of an improved patient outcome.

Peeling operations in highly myopic eyes (e.g., epiretinal membranes, foveoschisis, or full-thickness macula holes) could represent special indications where a robotic-assisted system may offer an additional benefit. The longer lever arm places special demands on the surgeon’s manual dexterity here, as movement artifacts come into play much more quickly.

The perception by the surgeons based on a survey questionnaire demonstrated that operating with the PSS was found to be more challenging than manual surgery. The surgeons had already performed hundreds of peelings manually and needed to learn how to perform the peeling using a joystick. However, the advantage for novice surgeons may be a steeper learning curve with the PSS than with manual surgery [10].

It is important to note that the learning curve may vary among surgeons and may be influenced by several factors, such as their experience, skill level, intensity of training, and willingness to learn new techniques. In particular, the fact that patients’ right eyes have to be operated by the surgeon’s right hand and vice versa pose extra challenges to the surgeons’ dexterity. Nevertheless, the survey questionnaire results provide valuable insights into the challenges surgeons face when operating with the PSS.

Our results must be considered in light of the limitations of this study. First, only nine patients received RA-MP with the PSS; thus, our cohort is relatively small. Two surgeons performed the surgeries alternately, which could lead to some differences. However, our study is rather explorative in nature, and both surgeons had comparable experience; therefore, we believe that these factors do not bias our work. Due to the low number of patients, a comparison to manually performed surgeries was not considered, although it is planned for the future. Moreover, the study addressed the integration of the PSS in the OR setting, focusing on preparation and surgical times, as well as the surgeon’s perception. Detailed information on the individual cases could not be provided, even if no severe PSS-related complications occurred.

Overall, the PSS seems to be an important and innovative surgical robot system for the field of vitreoretinal surgery. A larger, randomized, prospective study is warranted to further elaborate on the above aspects.

## Figures and Tables

**Table 1:** Definitions of the three main tasks of preoperative and operative steps and the associated subtasks. PSS = Preceyes Surgical System, IM = instrument manipulator, MC = motion controller, OR = operating room.

Main tasks	I. Preparation of PSS	II. Patient preparation	III. Surgical Intervention
	Start point: draping of IM, End point: head cushion placement	Start point: patient enters OR, End point: insertion of speculum	Start point: insertion of first trocar, End point: removal of last trocar
Subtasks	- Application of IM draping - Application of trocar holder - Application of extra sterile cover - Perform system test - Extra draping for MC/cables - Placement of black head cushion	- Anesthesia - Removal of all extra draping - Reachability check - Patient’s head fixation - Fellow eye draping and insertion of speculum	- Port and trocar placement - Vitrectomy - Chandelier twin light - Peeling procedure

**Table 2:** Four surgeries were performed by surgeon F.M.H., and five surgeries were performed by surgeon M.D.B.

Case	Surgeon
**1**	F.M.H.
**2**	M.D.B.
**3**	F.M.H.
**4**	M.D.B.
**5**	F.M.H.
**6**	M.D.B.
**7**	M.D.B.
**8**	F.M.H.
**9**	M.D.B.

**Table 3:** The mean times with ranges required for the three main tasks and the associated subtasks. * In case of combined surgery, the time needed for cataract surgery was excluded.

Main Task	Subtask I	Subtask II	Mean (Range) in Min
I. Preparation of PSSStart point: draping; end point: placement of head cushion			12.3 (6–15)
	Application of IM draping		
	Application of trocar holder		
	Application of extra sterile cover		
	Perform system test		
	Extra draping for MC/cables		
	Placement of black head cushion		
II. Patient preparation Start point: patient enters OR; end point: insertion of speculum			47.2 (36–65)
	Anesthesia Start point: patient enters OR; end point: patient is in general anesthesia		25.6 (9–55)
	Following Anesthesia Start point: removal of extra draping; end point: insertion of speculum	Removal of all extra draping	14.4 (2–21)
		Reachability check	
		Patient’s head fixation	
		Fellow eye draping and insertion of speculum	
III. Surgical intervention Start point: insertion of first trocar; end point: removal of last trocar *			72.4 (57–100)
	Port and trocar placement	Infusion trocar	2 (1–3)
		Nasal trocar	
		Temporal trocar/port	
	Vitrectomy Start point: insertion of vitrectomy cutter; end point: removal of vitrectomy cutter		7.9 (4–13)
	Chandelier twin light Start point: insertion of first chandelier; end point: insertion of second chandelier		3.4 (3–5)
	Peeling procedure Start point: bringing PSS in “on position”; end point: removal of forceps		41.8 (22–60)
		Connection IM and trocar	7.4 (2–18)
		If applicable: 2nd connection robot and trocar	4.9 (2–18)
		Membrane peeling start point: first grasp; end point: removal of forceps	27.9 (9–46)

## Data Availability

The data presented in this study are available on request from the corresponding author. The data are not publicly available due to confidentiality agreement with Carl Zeiss Meditec.
